# Epidemiological Features of Spinal Cord Injury in China: A Systematic Review

**DOI:** 10.3389/fneur.2018.00683

**Published:** 2018-08-22

**Authors:** Shiyang Yuan, Zhongju Shi, Fujiang Cao, Jiahe Li, Shiqing Feng

**Affiliations:** ^1^Department of Orthopaedics, Tianjin Medical University General Hospital, Tianjin, China; ^2^F.M. Kirby Neurobiology Center, Boston Children's Hospital and Department of Neurology, Harvard Medical School, Boston, MA, United States

**Keywords:** spinal cord injury, SCI, epidemiology, China, incidence

## Abstract

**Background:** Spinal cord injury (SCI) is a severe condition that disrupts patients' physiological, mental, and social well-being state and exerts great financial burden on patients, their families and social healthcare system. This review intends to compile studies regarding epidemiological features of SCI in China.

**Methods:** Searches were conducted on PubMed, EMBASE, Web of Science and Cochrane Library for relevant studies published through January, 2018. Studies reported methodological and epidemiological data were collected by two authors independently.

**Results:** Seventeen studies met the inclusion criteria. Two studies reported incidence of SCI that is 60.6 in Beijing (2002) and 23.7 in Tianjin (2004–2008). All studies showed male had a larger percentage in SCI compared to female except Taiwan (2000–2003). The average male and female ratio was 3–4:1 in China and the highest male and female ratio was 5.74: 1 in Tianjin (2004–2007). Farmers, laborers and unemployed people accounted for more than half of the SCI patients in China. Fall was the primary causation with exception of Heilongjiang (2009–2013), Beijing (2001–2010), and Taiwan (2002–2003), where motor vehicle collision (MCVs) was the leading causation. Pulmonary infection, urinary tract infection and bedsore were common complications, accounting for approximately 70% of SCI patients in China.

**Conclusion:** This review shows that epidemiological features of SCI are various in different regions in China and prevention should be implemented by regions. The number of patients with SCI result from fall and MCVs may become a main public health problem because population aging and economic developing in China. However, because all included studies were retrospective and lacking a register system in China, some data were incomplete and some cases may be left out, so the conclusion may not be generalizable to the other regions.

## Background

Spinal cord injury (SCI) is a devastating lesion, resulting in motor and sensory deficit and ultimately exerts impact on patient's physiological, mental and social well-being state (1, 2). Due to no effective treatment is available for SCI, unfortunately, SCI patients have to take substantial financial burden for their treatment and healthcare rehabilitation. Meanwhile, the incidence of SCI is rising worldwide with annual estimated incidence at 10.4–83 cases per million even though prevention measures have been taken to lower the occurrence (3, 4). The incidence of SCI range from 20.7 to 83.0 in North American and 8.0 to 130.6 in Europe per million annually (5, 6). Research have reported the incidence of SCI ranged between 10.6 and 22.6 per million in Québec, Canada (2000–2011) (7) and its annual costs was 2.67 billion dollars. In Denmark (1990–2012) (8) the incidence was 10.2 per million. And in Finland (2012–2013) (9) was 25.1–38.1 per million and 21.0–32.3 per million in Australia (1921–2011) (10). It has been estimated the costs of SCI in Australia annually to be almost 2 billion Australian dollars in total (11). In Japan, it has been reported the incidence of SCI was 121.4 per million in 2011 and 117.1 in 2012 (12). The financial burden of SCI includes rehabilitation services, expensive personal assistance, lost productivity for disability and social isolation. Studies have revealed the high incidence and heavy economic impact of SCI in developed countries.

As the largest developing country with approximately 25% of the global population, China has a large number of SCI patients. Unfortunately, little was known about the epidemiological features especially incidence and prevalence of SCI (13). Given the increasing life expectancy people with SCI are experiencing from 69 years in 1990 to nearly 75 years (14), prevention strategies and post-injury rehabilitation is crucial (15). Reliable epidemiological data and evidence of SCI in China are vital for estimating the number of patients, finding out the main causes, developing interventions, providing up-to-date information, and raising public awareness. Previous studies have reported the incidence of SCI in China increased significantly (16). Therefore, it is of great significance to review all the research data of SCI in China to attain a comprehensive understanding of its epidemiological features so as to improve management and reduce the financial burden on patients and healthcare system.

This review intends to compile the studies regarding epidemiological features of SCI published through January, 2018 in four directly governed cities–Beijing, Tianjin, Shanghai and Chongqing, and four provinces–Heilongjiang, Anhui, Guangdong and Taiwan. It will provide an updated overview for the epidemiological profile and give an insight into current situation of SCI in China. This can help us better carry out prevention strategies and effective allocate medical resources.

## Methods

**Figure 1:**
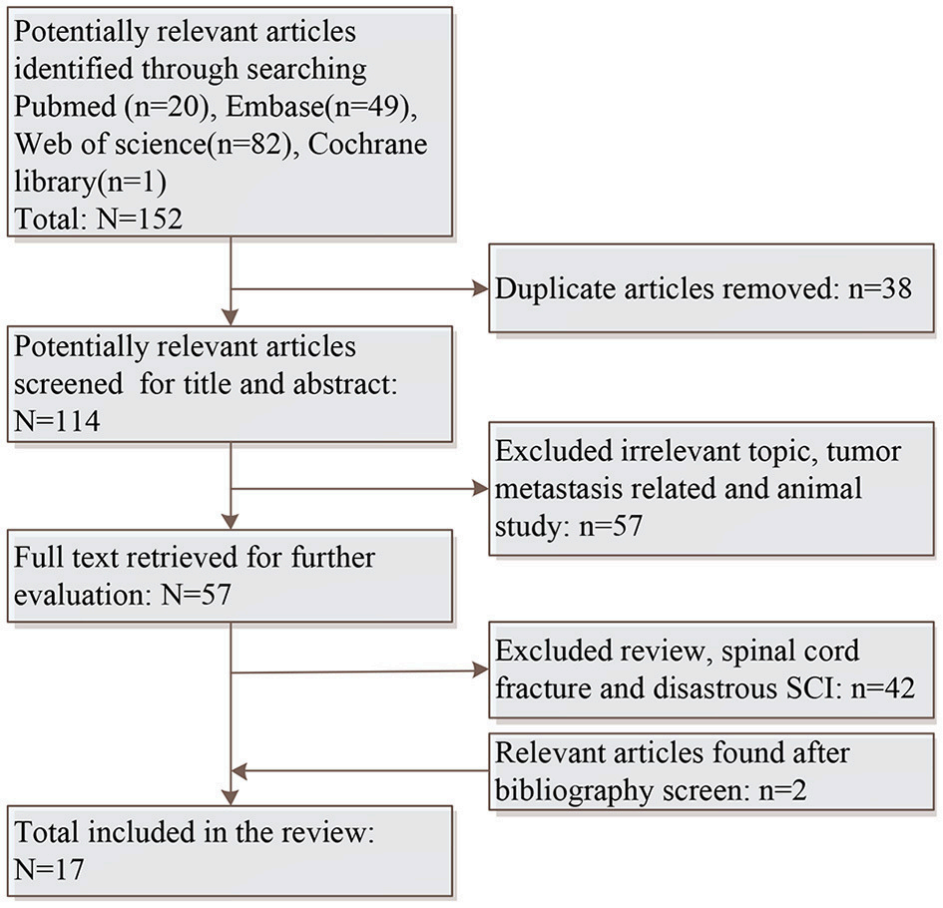
Flowchart of the systematic literature search.

Searches were conducted on PubMed, EMBASE, Web of Science and Cochrane Library for potentially relevant studies without language and date restrictions. The keywords and phrases applied for this search were “spinal cord injury,” “traumatic spinal cord injury,” “epidemiology,” and “China.” Bibliographies of included studies were also systematically screened to identify further relevant studies which were not included in the searched electronic databases. Case reports and conference abstracts were excluded. Figure 1 displays the search strategy and screen procedure in details.

Titles and abstracts were identified by two independent reviewers and were categorized by the previous inclusion criteria. The eligible inclusion criteria were presented as follow: (1) original study concerning SCI or TSCI; (2) studies provide relevant epidemiological data; (3) original data were collected from hospital. Results extraction was also conducted by two independent reviewers and all discrepancy were settled by discussing with a third reviewer. From included studies, methodological information and epidemiological data were collected: region, source population, incidence period, case criteria, study type (prospective or retrospective), total number of patients, incidence, causation, male and female ratio, mean age, age with peak incidence, age span, patients' occupation, injury level, injury extent, America Spinal Injury Association Impairment Scale (AIS) grade. Additionally, complication and treatment were extracted.

## Results

**Figure 2:**
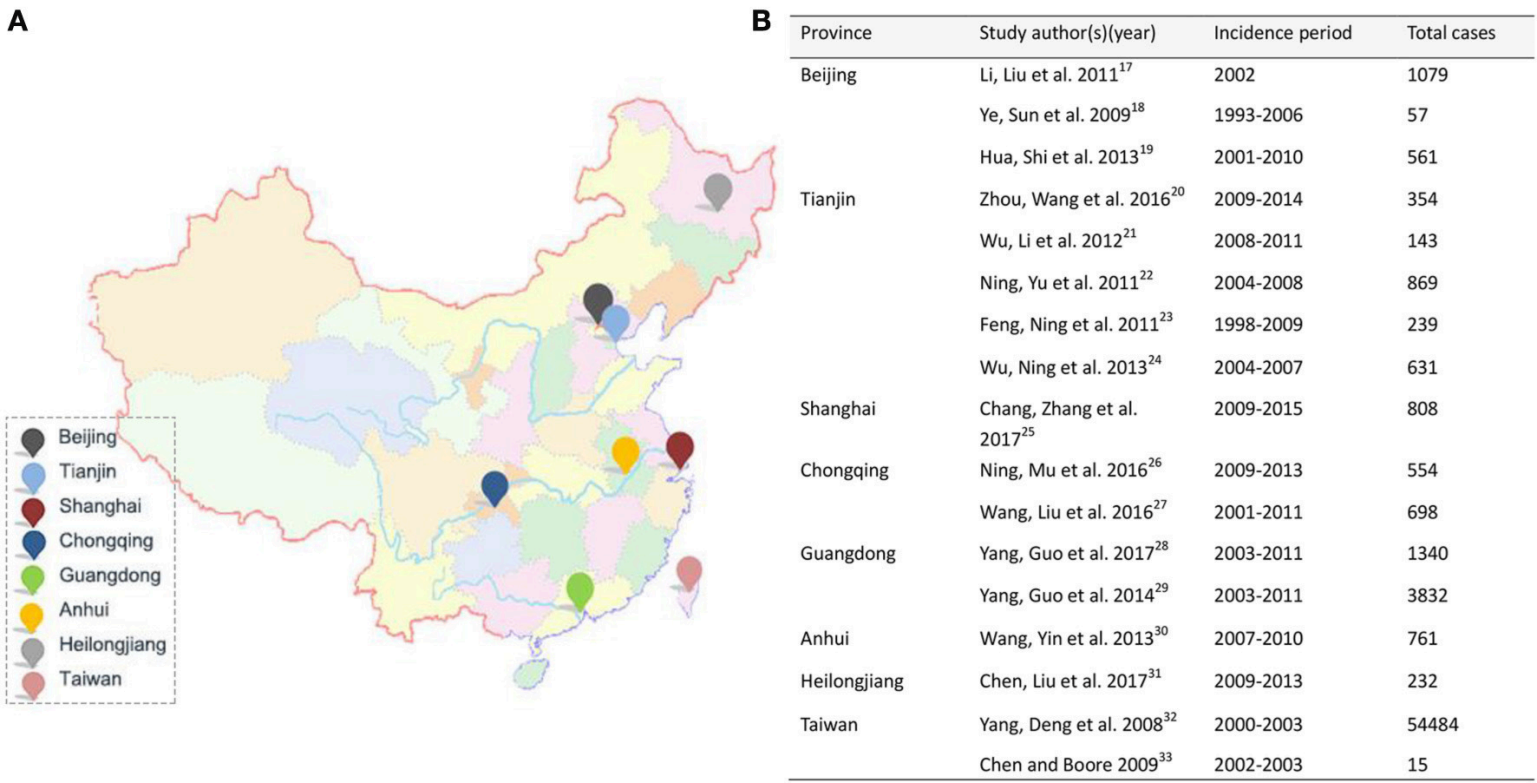
Regions **(A)** and literatures **(B)** are included in this study.

Total 152 potentially relevant studies were identified initially and 15 met the inclusion criteria. After systematically screening bibliographies of included studies, two additional were added. Therefore, a total of 17 studies were included finally. The epidemiological features of eight regions (Figure 2) were reported. Table 1 displays region, patient source, incidence periods, diagnostic criteria and study type, and number of patients, incidence, causation, male and female ratio, average age, peak incidence age and age span were listed in Table 2. Table 3 summarizes the occupation, causation, level of injury, ASI degree and treatment. Tables 4–8 present causation, occupation, segments, severity and complication, respectively.

**Table 1:** Characteristics of included studies of spinal cord injury in China.

**Study author (year)**	**Region**	**Incidence period**	**Patient source**	**Data source**	**Diagnostic criteria**	**Study type**
Li et al. (17)	Beijing	2002	TSCI patients admitted to hospital in Beijing	Records data from hospitals	Not reported	Retrospective
Ye et al. (18)^#^	Beijing	1993–2006	Patients in four hospitals and two rehabilitation institutions	Records data from hospitals	Not reported	Retrospective
Zhou et al. (19)	Tianjin	2009–2014	TSCI patients admitted to Tianjin medical university general hospital	Records data from hospitals	Not reported	Retrospective
Wu et al. (20)^##^	Tianjin	2008–2011	TCSCI patients aged ≥15 years admitted to a general hospital	Records data from hospitals	Diagnostic code ICD-10	Retrospective
Ning et al. (21)	Tianjin	2004–2008	TSCI patients aged 15 years or older admitted to tertiary hospitals in Tianjin	Records data from hospitals	ICD-10 codes for hospital admitted patients	Retrospective
Feng et al. (22)	Tianjin	1998–2009	All TSCI patients admitted to Tianjin medical university general hospital	Records data from hospitals	Diagnostic code T09.302	Retrospective
Chang et al. (23)	Shanghai	2009–2015	SCI individuals that enrolled in “halfway houses”	Data extracted from the Management Information System of the Shanghai Disabled Persons' Federation	Not reported	Retrospective
Ning et al. (24)	Chongqing	2009–2013	TSCI patients admitted to Xinqiao Hospital	Records data from hospital	International Classification of Diseases Version 10 (ICD-10) and diagnostic code of TSCI.	Retrospective
Yang et al. (25)	Guangdong	2003–2011	TSCI patients in second grade class-A hospitals	Records data from hospitals	International Classification of Disease Version 10 (ICD-10) and diagnostic code of TSCI.	Retrospective
Wang et al. (26)	Anhui	2007–2010	All TSCI patients admitted to two hospitals in Anhui province	Records data from hospitals	Not reported	Retrospective
Chen et al. (27)	Heilongjiang	2009–2013	TSCI patients in the Second Affiliated Hospital of Harbin Medical University and the Fifth Hospital of Harbin	Records data from hospital	Not reported	Retrospective
Yang et al. (28)	Taiwan	2000–2003	Patients hospitalized with spinal trauma in Taiwan from the National Health Insurance entire impatient database	Data from the nationwide National Health Insurance (NHI) database	ICD9-CM 806.00–806.9, 952.00–952.9	Retrospective
Chen and Boore 2009 (29)	Taiwan	2002–2003	Patients in a rehabilitation hospital in Taiwan	Semi-structured, tape recorded interview and observation of a group discussion	Not reported	Retrospective
Wang et al. (30)^###^	Chongqing	2001–2011	TSFs patients admitted to Third Military Medical University affiliated hospitals	Records data from hospitals	Not reported	Retrospective
Wu et al. (31)	Tianjin	2004–2007	TSCI patients admitted to 17 tertiary hospitals in Tianjin	Records data from hospitals	Not reported	Retrospective
Hua et al. (32)	Beijing	2001–2010	TSCI patients admitted to the General Hospital of Chinese People's Armed Police Forces	Records data from hospital	version 1.2 of the International Classification of External Causes of Injuries	Retrospective
Yang et al. (33)	Guangdong	2003–2011	Patients admitted to the sampled hospitals in Guangdong	Records data from hospitals	Not reported	Retrospective

# *sports related SCI*;

## *cervical spinal cord injury*;

### *SCI resuiting from motor vehicle collisions*.

*ICD, International Classification of Disease; TSCI, traumatic spinal cord injury; TSF, traumatic spinal fracture*.

**Table 2:** Incidence, cause, gender ratio and age of spinal cord injury in China.

**Study author (year)**	**Region**	**Incidence period**	**Total case**	**Incidence**	**Leading cause**	**Second cause**	**Gender ratio**	**Average age (years)**	**Peak age (years)**	**Age range**
Li et al. (17)	Beijing	2002	1, 079	60.6	Fall (41.3%)	MVCs (22.3%)	3.1:1	Overall: 41.7	30–39 (36.2%)	6–80
Ye et al. (18)^#^	Beijing	1993–2006	57	N	Water sports (64.9%)	Gymnastics (8.8%)	3.3:1	Overall: 24.49 ± 11.92	12–29 (63.2%)	5–58
Zhou et al. (19)	Tianjin	2009–2014	354	N	Fall (55.1%)	MVCs (35.9%)	2.34:1	Male: 49.1 ± 15.4 Female: 52.3 ± 15.6	46–60 (40.4%)	N
Wu et al. (20)^##^	Tianjin	2008–2011	143	N	Fall (49.7%)	MVCs (36.4%)	5:1	Male: 53.5 ± 14.9 Female: 54.2 ± 12.1	55–64 (29.4%)	18–87
Ning et al. (21)	Tianjin	2004–2008	869	23.7	Fall (56.9%)	MVCs (34.1%)	5.63:1	Male: 45.8 ± 14.2 Female: 47.5 ± 14.5	46–60 (39.4%)	16–90
Feng et al. (22)	Tianjin	1998–2009	239	N	Fall (41.9%)	MVCs (36.4%)	4.6:1	45.4 ± 14.1	45–54 (26.8%)	N
Chang et al. (23)	Shanghai	2009–2015	808	N	Traumatic injury (58.0%)	Disease (29.5%)	2.1:1	N	46–60 (30.3%)	N
Ning et al. (24)	Chongqing	2009–2013	554	N	Fall (61.7%)	MVCs (21.8%)	4.33:1	Male: 45.7 ± 13.3 Female: 45.4 ± 15.7	31–45 (36.3%)	4–81
Yang et al. (25)	Guangdong	2003–2011	1, 340	N	Fall (42.5%)	MVCs (37.8%)	3.5:1	41.6 ± 14.7	41–60 (43.0%)	1–84
Wang et al. (26)	Anhui	2007–2010	761	N	Fall (65.3%)	Transport (21.2%)	3.4:1	Overall: 45	31–45 (42.6%)	N
Chen et al. (27)	Heilongjiang	2009–2013	232	N	MVCs (42.7%)	Fall (36.2%)	4:1	Male: 45.07 ± 14.1 Female: 46.5 ± 15.40	46–60 (36.6%)	18–82
Yang et al. (28)	Taiwan	2000–2003	19, 007	N	N	N	N	N	N	N
Chen and Boore 2009 (29)	Taiwan	2002–2003	15	N	MVCs	Fall	2.75:1	Overall: 31	N	N
Wang et al. (30)^###^	Chongqing	2001–2011	298	N	MVCs	N	N	N	N	N
Wu et al. (31)	Tianjin	2004–2007	631	N	Fall (56.6%)	MVCs (34.4%)	5.71:1	Male: 31.6 ± 37.0 Female: 37.3 ± 41.5	46–60 (37.9%)	N
Hua et al. (32)	Beijing	2001–2010	561	N	MVCs (51.2%)	Fall (23.9%)	4.1:1	Overall: 34.74 ± 12.24	N	0.75–47
Yang et al. (33)	Guangdong	2003–2011	3, 832	N	MCVs (21.7%)	Strike by objects (19.5%)	3.0:1	Overall: 42.4 ± 15.3	41–60 (36.4%)	1–94

# *sports related SCI*;

## *cervical spinal cord injury*;

### *SCI resulted from motor vehicle collisions*.

*N, not reported; Gender ratio, M/F; MVC, motor vehicle collision; Incidence, million/year*.

**Table 3:** Occupation, causation, level and extent of injury, AIS and treatment of spinal cord injury in China.

**Study author (year)**	**Region**	**Occupation**	**Causation**	**Level of injury**	**Complete/tetraplegia**	**AIS**	**Treatment**
Li et al. (17)	Beijing	Worker: 29.9% Farmer: 32.6% Clerical staff: 5.7% Government officials: 1.5% Others: 30.3%	Fall: 41.3% Traffic: 22.3% Struck: 18.6% Sports: 1.1% Stab: 0.4% Others: 16.3%	Cervical: 4.9% Thoracic: 28.0% Thoracolumbar, lumbar, lumbosacral: 66.0%	N	N	Surgery: 36.7% Conservative: 63.3%
Ye et al. (18)^#^	Beijing	N	N	N	54.5%/89.0%	A: 56.1% B: 33.3% C: 8.8% D: 1.8%	N
Zhou et al. (19)	Tianjin	Peasants: 32.5% Workers: 24.3% Unemployed: 16.9% Office clerks: 7.3% Retired: 11.9% Teachers: 2.0% Students: 1.7% Drivers: 3.4%	MVCs: 35.9% Low fall: 33.6% High fall: 21.5% Falling objects: 2.8% Collide head: 0.8% Machine: 0.6% Sports: 2.8% Massage: 0.6% Others: 1.4%	Cervical: 71.5% Thoracic: 13.3% Lumbar and sacral: 15.2%	20.9%/67.2%	A: 20.9% B: 11.3% C: 20.9% D: 47.9%	Surgery: 57.6% Conservative: 32.8% Other: 9.6%
Wu et al. (20)^##^	Tianjin	N	Low fall: 45.5% High fall: 4.2% MVA: 36.4% Work accident: 2.1% Sport-related: 4.2% Struck by object: 4.9% Assault: 1.4% Other: 1.4%	N	N	A: 5.6% B: 16.8% C: 18.9% D: 58.7%	N
Ning et al. (21)	Tianjin	N	MVC: 34.1% Low fall: 37.6% High fall: 19.3% Struck by object: 6.3% Sport-related: 0.2% Assault: 1.4% Work accident: 0.8% Other: 0.2%	Cervical: 71.5% Thoracic: 13.3% Lumbar: 15.1% Sacral: 0.1%	25.2%/71.6%	A: 25.2% B: 18.2% C: 14.7% D: 41.9%	N
Feng et al. (22)	Tianjin	Worker: 15.1% Peasant: 20.1% Driver: 4.2% Unemployed: 48.5% Retired: 5.0% Civil servants: 3.8% Teachers: 2.1% Students: 1.2%	MVCs: 36.4% Low fall: 35.2% High fall: 16.7% Falling objects: 5.4% Collision of head: 2.5% Machine: 2.1% Sports: 0.4% Massage: 0.4%	Cervical: ≥80.0%	32.6%/82.4%	A: 32.6% B: 12.1% C: 16.3% D: 38.9%	N
Chang et al. (23)	Shanghai	N	Congenital: 4.1% Disease: 29.5% traumatic injury: 58.0% other: 8.4%	N	46.7%/N	N	Surgery: 53.8% Medicine: 49.0% Rehibilitation training: 46.9% Assistive devices: 41.1% Physical therapy: 21.3% Traditional therapy: 17.0% Other: 17.7%
Ning et al. (24)	Chongqing	Laborer: 23.6% Peasant: 32.1% Unemployed: 24.2% Office-clerks: 3.6% Retired: 2.0% Teacher: 0.4% Student: 1.8% Other: 12.5%	MVCs: 21.8% Low fall: 10.8% High fall: 50.9% Falling objects: 13.2% Collision of head: 0.2% Machine: 0.9% Sports: 0.5% Hyper-traction:0.4% Other: 1.2%	Cervical: ≥54.0%(C4–C6:36.8%) Thoracolumbar (T11–T12:30.3%)	39.3%/54.9%	A: 39.4% B: 8.7% C: 21.1% D: 30.8%	Surgery: 83.9% Conservative: 13.5% Other: 2.5%
Yang et al. (25)	Guangdong	Workers: 5.1% Peasants: 10.4% Unemployed: 0.7% Self-employed: 0.7% Enterprise staff: 2.1% Civil servants: 1.9% Teachers: 8.5% Retired: 1.0% Students: 44.3% Other: 25.4%	Falling objects: 8.8% Violence: 8.7% High fall: 41.0% Traffic accidents: 37.8% Crushing injuries: 1.0% Sports: 1.3% Low fall: 1.5%	Cervical: 61.0% Thoracic: 20.5% Lumbar: 22.8%	26.4%/N	N	Surgery: 35.4% Rehabilitation: 34.7% Traction: 17.5% Hyperbaric oxygen: 11.9%
Wang et al. (26)	Anhui	Farmer: 57.2% Laborer: 13.3% Student: 2.6% Civil servant: 3.4% Others: 12.4%	Transport: 21.2% Struck by object: 5.4% Fall from height: 52.6% Low fall: 12.7% Others (assault, sport-related injury): 8.1%	Cervical: 46.3% Thoracic: 20.4% Lumbosacral: 33.3%	N	A: 25.6% B: 11.8% C: 27.3% D: 35.2%	Surgery: 71.0% Conservative: 12.7% Other: 15.8%
Chen et al. (27)	Heilongjiang	Farmer: 35.34% Workers: 10.78% Civil servants: 15.95% Students: 6.47% Retired: 21.98% Othera: 9.48%	Motor vehicle collisions: 42.7% High fall: 13.8% Low fall: 22.4% Collision of head: 7.3% Blunt-force trauma: 3.0% Falling objects: 3.8% Pierce: 2.2% Sport-related: 5.2%	Cervical: 76.29% Cervicothoracic: 3.88% Thoracic: 10.34% Thoracicolumba: 4.74% Lumbosacral: 4.74%	18.17%/76.36%	A: 14.22% B: 15.09% C: 32.76% D: 37.93%	Surgery: 44.0%
Yang et al. (28)	Taiwan	N	N	N	N	N	N
Chen and Boore 2009 (29)	Taiwan	N	N	N	46.7%/80.0%	N	N
Wang et al. (30)^###^	Chongqing	N	N	N	34.9%/N	N	N
Wu et al. (20)	Tianjin	N	MVC: 34.4% Low fall: 38.7% High fall: 17.9% Struck by object: 6.3% Others: 2.7%	Cervical: 71.2% Thoracic: 13.0% Lumbar: 15.8%	N	A:21.6% B:20.9% C:17.3% D:40.3%	Surgery: 53.7%
Hua et al. (32)	Beijing	N	Transportation accident: 51.2% Falling from a height: 23.9% Tamping: 8.6% Stumbling: 8.0% Stabbing: 3.0% Crushing: 1.8% Gunshot wound: 1.1% Diving: 1.1% Spinal surgery: 0.7% Blunt-force trauma: 0.4% Traction by machine: 0.4%	Cervical: 47.2% Cervicothoracic: 3.4% Thoracic: 33.2% Thoracicolumbar: 7.1% Lumbosacral: 5.5%	49.9%/N	N	N
Yang et al. (33)	Guangdong	Workers: 36.2% Peasants: 22.8% Unemployed: 13.9% Retired: 6.9% Other occupations: (self-employed, civil servants, students, enterprise staff): 9.6% Other: 10.5%	Traffic accidents: 21.7% Struck by objects: 19.5% Crushing injuries: 15.1% High falls: 9.8% Non-traumatic:8.2% Unknown:18.1% Other:7.7%	Cervical: 44.9% Thoracic: 33.0% Lumber: 24.6%	40.7%/N	N	Surgery: 55.4% Rehabilitation: 21.7% Traction: 12.8% Hyperbaric oxygen therapy: 7.2%

# *sports related SCI*;

## *cervical spinal cord injury*;

### *SCI resulted from motor vehicle collisions*.

*AIS, ASIA Impairment Scale; N, not reported; MVC, motor vehicle collision*.

**Table 4:** Causes of spinal cord injury in China.

**Study author (year)**	**Province**	**Low fall**	**High fall**	**MVCs**	**Struck by object**	**Sport related**	**Work accident**	**Assault**	**Others**
Li et al. (17)	Beijing	A1	A2	59 (22.3%)	49 (18.6%)	3 (1.1%)	N	1 (0.4%)	43 (16.3%)
Zhou et al. (19)	Tianjin	119	76	127	10	10	N	5	7
Wu et al. (20)^##^	Tianjin	65 (45.5%)	6 (4.2%)	52 (36.4%)	7 (4.9%)	6 (4.2%)	3 (2.1%)	2 (1.4%)	2 (1.4%)
Ning et al. (21)	Tianjin	327 (37.6)	168 (19.3)	296 (34.1)	55 (6.3)	2 (0.2)	7 (0.8)	12 (1.4)	2 (0.2)
Feng et al. (22)	Tianjin	35.2%	16.7%	36.4%	5.4%	0.4%	N	N	5%
Ning et al. (24)	Chongqing	60	282	121	73	3	N	N	15
Yang et al. (25)	Guangdong	20 (1.5%)	594 (41.0%)	506 (37.8%)	188 (8.8%)	18 (1.3%)	N	116 (8.7%)	13 (1.0%)
Wang et al. (26)	Anhui	97 (12.7%)	400 (52.6%)	161 (21.2%)	41 (5.4%)	N	N	N	62 (8.1%)
Chen et al. (27)	Heilongjiang	52	32	99	8	12	N	N	29
Wu et al. (31)	Tianjin	244 (38.7%)	133 (17.9%)	217 (34.4%)	40 (6.3%)	N	N	N	17 (2.7%)
Hua et al. (32)	Beijing	N	134 (23.9%)	287 (51.2%)	N	N	N	17 (3.0%)	123 (22.1%)
Yang et al. (33)	Guangdong	B1	B2	831 (21.7%)	746 (19.5%)	N	N	N	1,880 (49.1%)

## *cervical spinal cord injury; A1 + A2 = 109 (41.3%); B1 + B2 = 374 (9.8%)*.

*N, not reported; MVCs, motor vehicle collisions*.

### Incidence, gender, and age

In this review, only two studies reported incidence of SCI, in Beijing (2002) and Tianjin (2004–2008), which is 60.6 and 23.7 per million, respectively (17, 21). Totally 1,948 cases was included. We found incidence varied significantly, the highest was 60.6 in Beijing, which was approximately three times as much as Tianjin (2004–2008).

All studies showed male had a larger percentage of SCI compared to female The male and female ratio ranged significantly across regions, with the highest ratio was 5.71: 1 in Tianjin (2004–2007) (31). In Tianjin (2004–2008, 5.63:1; 2008–2011, 5:1), male and female ratio was high, while in Shanghai (2009–2015), male and female ratio was 2.1:1, which was ralitively lower than other regions. And in China the average male: female ratio was 3–4:1.

As expected, the mean age was range from 30 to 50 typically, the average age of male was slightly younger than that of female among reported studies except Chongqing (2009–2013) (24), where male and female had an average age of 45.7 and 45.4, respectively. Age with peak incidence ranged from 40 to 60 years. However, Beijing (2002) had a relative lower age of peak incidence which was 30–39 years of age (17). Both Chongqing (2009–2013) and Anhui (2007–2010) had a peak incidence aged 31–45 years (24, 26). The age span ranged a broader spectrum, the youngest was 9 months in Beijing (2001–2010) while the oldest was 90 years in Tianjin (2004–2008) (21, 32).

### Occupations

As shown in Table 5, the occupations of SCI patients varied including the farmers, laborers, civil servants, office clerks, students, teachers, retired, unemployed and others. Classification of their occupations on the basis of their workplace. It was evident that farmers, laborers and unemployed people had a higher risk of SCI. These three groups accounted for more than half of the patients with SCI. In Beijing (2002) and Anhui (2007–2010), manual workers like farmers and peasants have a high ricks than Guangdong (2003–2011) and Heilongjiang (2009–2013). Surprisingly, teachers (8.5%) and students (44.3%) with SCI in Guangdong (2003–2011) outnumbered peasants (10.4%) and workers (5.1%) (25). Additionally, retired people with SCI taken up a large percentage in Heilongjiang (2009–2013: 21.98%), which exceeded the number of workers (10.78%) (27).

**Table 5:** Occupations of spinal cord injury in China.

**Study author (year)**	**Province**	**Total cases**	**Farmer or peasant**	**Worker or laborer**	**Civil servants or office workers**	**Students or teacher**	**Retired or unemployed**	**Others**
Li et al. (17)	Beijing	1,079	86 (32.6%)	79 (29.9%)	19 (7.2%)	N	N	80 (30.3%)
Zhou et al. (19)	Tianjin	354	115	86	26	13	102	12
Feng et al. (22)	Tianjin	239	20.1%	15.1%	3.8%	3.3%	53.5%	4.2%
Ning et al. (24)	Chongqing	554	177	131	20	12	145	69
Yang et al. (25)	Guangdong	1,340	139 (10.4%)	68 (5.1%)	53 (4%)	708 (52.8%)	23 (1.7%)	349 (26.1%)
Wang et al. (26)	Anhui	761	435 (57.2%)	186 (13.3%)	26 (3.4%)	20 (2.6%)	N	94 (12.4%)
Chen et al. (27)	Heilongjiang	232	82 (35.34%)	25 (10.78%)	37 (15.95%)	15 (6.47%)	51 (21.98%)	22 (9.48)
Yang et al. (33)	Guangdong	3,832	873 (22.8%)	1,387 (36.2%)	N	N	798 (20.8%)	774 (20.1%)

*N, not reported*.

### Causes

With respect to causes of SCI, this study summarized the common cause in Table 4. Motor vehicle collisions (MCVs), high and low fall, struck by falling object, sports related activities, violence such as gunshot and stab, work related accidents, crushing injuries and blunt force trauma, others such as massage and collision of head. Among all these causes, fall including high and low fall were the primary causation, with the exception of few regions, such as Heilongjiang (2009–2013), Beijing (2001–2010), and Taiwan (2002–2003), where MCVs was the leading causes typically (27, 29, 32). The percentage of spinal cord injuries caused by struck by falling object was relative high in Guangdong (2003–2011, 19.5%), Beijing (2002, 18.6%), and Chongqing (2009–2013, 13.2%) than other regions (17, 24, 33). In Guangdong, however, injuries caused by violence taken up a larger percentage (8.7%). Details of sports and work related SCI was only reported in a few studies.

### Level and severity

Tables 6,7 present the injury level of SCI. Cervical spinal cord injuries taken up 45–70% in most regions, with the exception of Beijing (2002, cervical: 4.9%) (17), and the most common anatomical level was C4–C6. In Beijing (2002), the proportion of low level SCI like thoracolumbar, lumbar and lumbosacral injury was 66.0%, which is much higher than cervical (4.9%) and thoracic (28.0%) injury.. In addition, some patients injuried more than one level of spinal cord in Heilongjiang (2009–2013), where people subjected to cervicothoracic (3.88%), thoracicolumbar (4.74%) and lumbosacral (4.74%) injury (27).

**Table 6:** Level of spinal cord injury in China.

**Study author (year)**	**Province**	**Cases**	**Cervical**	**Thoracic**	**Lumber or sacral**
Li et al. (17)	Beijing	1,079	13 (4.9%)	74 (28.0)	176 (66.0%)
Zhou et al. (19)	Tianjin	354	238	62	54
Ning et al. (21)	Tianjin	869	621 (71.5%)	116 (13.3%)	132 (15.2)
Yang et al. (25)	Guangdong	1,340	818 (61.0%)	275	306
Wang et al. (26)	Anhui	761	352 (46.3%)	155 (20.4%)	254 (33.3%)
Chen et al. (27)	Heilongjiang	232	177 (76.3%)	24 (10.3%)	4.74%
Wang et al. (30)^###^	Chongqing	698	310 (30.1%)	600 (58.2%)	121 (11.7%)
Wu et al. (31)	Tianjin	631	449 (71.2%)	82 (13.0%)	100 (15.8%)
Yang et al. (33)	Guangdong	3,832	1,720 (44.9%)	1,264 (33.0%)	941 (24.6%)

### *SCI resuiting from motor vehicle collisions*.

**Table 7:** AIS of spinal cord injury in China.

**Study author (year)**	**Province**	**Total cases**	**AIS (A)**	**AIS (B)**	**AIS (C)**	**AIS (D)**
Ye et al. (18)^#^	Beijing	57	32 (56.2%)	19 (33.3%)	5 (8.8%)	1 (1.8%)
Zhou et al. (19)	Tianjin	354	74	40	74	166
Wu et al. (20)^##^	Tianjin	143	8 (5.6%)	24 (16.8%)	27 (18.9%)	84 (58.7%)
Ning et al. (21)	Tianjin	869	219 (25.2%)	158 (18.2%)	128 (14.7%)	364 (41.9%)
Feng et al. (22)	Tianjin	239	32.6%	12.1%	16.3%	38.9%
Ning et al. (24)	Chongqing	554	218 (39.4%)	48 (8.7%)	117 (21.1%)	171 (30.8%)
Wang et al. (26)	Anhui	761	195 (25.6%)	90 (11.8%)	208 (27.3%)	268 (35.2%)
Chen et al. (27)	Heilongjiang	232	33 (14.2%)	35 (15.1%)	76 (32.8%)	88 (37.9%)
Wu et al. (31)	Tianjin	631	136 (21.6%)	132 (20.9%)	109 (17.3%)	254 (40.3%)

# *sports related SCI*;

## *cervical spinal cord injury*.

*AIS, America Spinal Injury Association Impairment Scale*.

The severity of SCI is measured by neurological degree (tetraplegia and paraplegia) and extent (complete and incomplete). In the present review, 6 studies reported degree and 11 studies reported extent. Tetraplegia injury had a large proportion than paraplegia in all included studies and the proportion of tetraplegia patients ranged between 54.9 and 89.0%. The largest proportion was in Beijing (1993–2006) and smallest in Chongqing (2009–2013), respectively (18, 24). Regarding extent of injuries, the proportion of complete injuries varied from 18.17 to 54.5% and the percentage of incomplete injuries great than complete injuries. In Beijing (1993–2006), the occurrence of tetraplegia and complete injury was much higher than Chongqing (2009–2013), Guangdong (2003–2011) and Heilongjiang (2009–2013).

The AIS grade was reported in 9 studies. In included studies, most patients were grade D except Beijing (1993–2006) and Chongqing (2009–2013) (18, 24), which grade A was the most. In Beijing (1993–2006), grade A patients accounted for 56.1%, which was higher than that in Chongqing (2009–2013). Patients of grade B and grade C maintained a middle position, constituted approximately 10–40% combined.

Seven studies reported the number of death. Tianjin (2008–2011) had high death rate (4.2%) among all regions (20). Patients aged over 60 and with complications are more vulnerable to death during their hospitalization.

### Clinical complications and treatment

In this review, six studies reported clinical complications in Table 8. Pulmonary infection, urinary tract infection and bedsore are the most common complications which was approximately 70%, followed by deep venous thrombosis and electrolyte disturbance. Surprisingly, patient with hyponatremia in Tianjin (2008–2011) was 30.1%, which was nearly twice as much as patients with respiratory infection (18.2%) (20). In Guangdong (2003–2011), rare complications were reported, such as urinary calculus (0.3%), spasms (0.3%), and autonomic dysreflexia (0.9%) (25).

**Table 8:** Complications of spinal cord injury in China.

**Study author (year)**	**Province**	**Total cases**	**Pulmonary infections**	**Urinary tract infections**	**Bedsores**	**Venous thrombus**	**Hyponatremia**
Zhou et al. (19)	Tianjin	354	2.8%	1.7%	0.8%	3	N
Wu et al. (20)^##^	Tianjin	143	26 (18.2%)	33 (23.1%)	14 (9.8%)	7 (4.9)	43 (30.1%)
Yang et al. (25)	Guangdong	1,340	123 (36.5%)	97 (28.8%)	52 (15.4%)	12 (3.6%)	22 (6.5%)
Wang et al. (30)^###^	Chongqing	298	11	N	6	3	N
Wu et al. (31)	Tianjin	631	51 (8.3%)	33 (5.2%)	17 (2.7%)	10 (1.6%)	28 (4.4%)
Yang et al. (33)	Guangdong	3,832	185 (37.6%)	129 (26.3%)	67 (13.6%)	14 (2.8%)	51 (10.3%)

## *cervical spinal cord injury*;

### *SCI resuiting from motor vehicle collisions*.

*N, not reported*.

The main treatment for SCI was surgery, including decompression and internal fixation or fuse. In the included studies, more than half of the SCI patients received surgery. Nevertheless, a larger proportion of patients (63.3%) in Beijing (2002) received conservative treatment than surgery (36.7%) (17). In addition, a much larger percentage of patients in Guangdong (2003–2011, 34.7%) received rehabilitation than other regions in China (25). Rehabilitation strategy including braces can be used to practice standing and walking, and other special tools, such as walking aids, can be provided to compensate for the lack of moter funcion.

## Discussion

SCI impairs the physical, metal, and social well-being state of patients and exerts heavy financial burden on national healthcare system, patients and their families. A thorough understanding of SCI epidemiological profile helps national healthcare system carry out preventative strategies better and allocate medical resource reasonablely. Also, a comparison between different cities and provinces can shed light on how to effectively tackle these issues in the process of implementing prevention measures.

Many studies of SCI were carried out in developed nations, especially in America, Canada and Australia. Developing countries, however, with approximately 80% population throughout the world, lack related data because a national register system for SCI has not been established yet. To the best of our knowledge, several reviews of SCI in China have been reported. In China, it is difficult to estimate incidence because universal diagnostic criteria and national register system were not established. In this study, only 2 studies reported estimated incidence. In 2002, the incidence in Beijing was 60.6 per million, while it was 23.7 per million in Tianjin (2004–2008) (21, 28). Studies in China used various inclusion criteria, so the incidence varied significantly. In developed countries, the annual incidence was 20.7–83 per million in America and 8.0–130.6 in Europe (34). This indicate the incidence of SCI in China was similar with developed countries. And other studies have reported developing regions had lower incidence compared to developed countries (35) and such difference might due to the development level of society and economy. In Europe and American, people are more likely to participate in risky activities like housing riding, rugby and skiing and these are high risk factors of SCI. In China, ages with peak incidence is different from other countries. Ages with peak incidence was varied from 30 to 60 years, while it was 20–29 in Aragon, Spain (1972–2008) and 16–31 in Thessaloniki, Greece in 2006 (36, 37). In Beijing (1993–2006), peak age of patient was 12–29, which is younger than other studies because it focud on sports related SCI and most athletes were young people. In addition, the mean age of SCI in North America was range from 32 to 55.4 years (38), and it was between 37 and 47.9 in Europe (39). This was in consistence with mean age in China, which is from 30 to 50. Demographic structure is a main factor to explain varied ages with peak incidence across different countries. In China, the ratio of old people is increasing dramatically. It has been predicted individual aged over 60 could be 438 million by 2050 (40). Old people are more vulnerable to SCI due to degeneration of vertebrae and deterioration of physical conditions, so prevention is of great importance. The elderly should aware of the potential risks and take precautionary measures, such as do not do strenuous exercise and go to hospital regularly for physical examinations.

Most studies showed that male were at higher risk of SCI than female (41, 42), these results were in accordance with present study in China. The ratio up to 5.71:1 in Tianjin (2004–2207). This was ascribed to the Chinese traditional cultural backgrounds. Male are major sources of household income and take the responsibility to support their families while female have to stay at home and take care of children. Meanwhile, male are the main labor resources and productive group of society and many engage in manual work. Thus, a large number of male work on high risk industry and thay are more likely to be exposed to risky environment, such as industrial construction sites. However, with the progress of society, more female are taking high risk occupation, it could cause a slightly increase of female patients with SCI. In Beijing and Shanghai, the ratio was relatively lower because male and female nearly have equal access to various jobs in big cities. It was reported in Yang et al. (25) that the mean age of SCI patients was 41.6 (14.7) and age group of 41–60 had most injuries, but this was inconsistent with the high percentage of students (44.3%), which was much higher than worker (5.1%), and we can not explain this inconsistency.

Traffic accidents and falls are main causes of SCI in developed countries (43–45). People in these countries usually have a private car and this cause a high rate of car accidents. Old people living alone are prone to fall when walking up and down stairs. Similarly, MVCs and fall were the two leading cause in different regions in China (40). With the development of economy in China, most people had a private car and this led to the significant increase of car accidents, and disabilities caused by traffic accidences can be reduced through improve vehicle safety, driver behavior and road conditions (46). MCVs in Heilongjiang, Taiwan, Chongqing (2001–2011), Beijing (2001–2010), Guangdong (2003–2011) is the most common cause, while fall is the leading cause in Beijing (2002), Tianjin (2009–2014, 2008–2011, 2004–2008, 1998–2009, 2004–2007), Chongqing (2009–2013), Guangdong (2003–2011) and Anhui (2007–2010). In Beijing (2002), the study reported fall was the leading cause, while in 2001–2010, in 10-year-period, Beijing had development a lot and increasingly number of people owned their private car. Rate of car accident increased and MCVs became the leading cause. In Chongqing (2001–2011), the study was focused on SCI resulted from MCVs, while in 2009–2013, the leading cause was fall. Chongqing is called “mountain city” in China and the city is in mountains, so the incidence of fall was high. In Guangdong, Yang et al. (25) reported fall was leading cause, while Yang et al. (33) reported MCVs was the most common cause. These 2 studies based on different populations, so they got different conclusion. In Yang et al. (25) included 1340 cases, while 3,832 cases in Yang et al. (33). Low fall resulted in a large proportion of SCI as people aging. With social progresses and lifestyle changes, increasing number of old people pay more attention to their health conditions and engage in various activities, lacking of protective measures and experiences could increase risks of SCI. In some Western Asia countries, gunshots and violent conflicts account for a large proportion of SCI because people are accessible to firearms (47–49).

SCI can cause sensory or motor deficit and bladder dysfunction, so patients have high risks of complications. Pulmonary infection, urinary tract infection (UTIs) and bedsore are common complications, accounting for approximately 70%. Pulmonary infection in Guangdong (2003–2011) was high, which (33) and nearly twice as high as Tianjin (2008–2011). This because patients with high leval cervical SCI require mechanical ventilation but poor management will develop ventilator associated pneumonia. Besides, ability to cough is impaired due to paralysis of breathing muscles and it can cause pulmonary infections. UTIs in SCI patient are high both in high-income countries and less developed countries (50, 51) because patients who have bladder dysfunction have to use catheterization as a management method. The incidence of UTIs was high in Tianjin (2008–2011) and Guangdong (2003–2011). Evidences have shown that the methods of bladder management and different catheter material might relavant to UTIs (50). Other factors such as personal hygiene and long-term apply of indwelling catheter are associated with UTIs. Education on catheterization techniques and care can reduce and avoid UTIs. In addition, patients with SCI are more likely to suffering from bedsore due to sensation and mobility impair. Patients with bedsores were high in Guangdong (2003–2011, 15.4%) and Tianjin (2008–2011, 9.8%) (20, 25). Hospitals and rehabilitation centers should provide patients with careful management to prevent it, such as turn over frequently and skin checks on daily basis. Meanwhile, patients and their family members need training for nursing techniques to avoid bedsore. Furthermore, patients with SCI easily develop deep venous thrombosis (DVT). The percentage of DVT was reported in Tianjin (2008–2011) and in Guangdong (2003–2011), which needs rapid antithrombotic therapy to prevent potential pulmonary embolism. Electrolyte disturbance can be caused by methylprednisolone administration. It is controversial to use methylprednisolone currently but high dose use within 8 h after SCI is considered as a treatment option in China (52).

Studies have showed that surgery is the main treatment especially for complete injuries because it can prevent further injuries and improve spinal cord conditions (17, 19, 23, 25–27, 33). But weather perform surgery was depend on the severity and extent of injuries. Moreover, some patients chose conservative therapy while others against medical advice and left. Importantly, long term rehabilitation therapy is necessary for further functional recovery. Physical therapy is the main rehabilitation therapy and it based on functional training like standing and walking practice. Nursing and medication are also used to prevent complications in rehabilitation centers. Rehabilitation center was advanced in high income countries, whereas it is not well built in China.

Despite increasing number of literature was published, it was little research that summarize all the studies so as to further investigate the epidemiological features of SCI in China. This review tries to provide a more detailed epidemiological profile of SCI in China, but challenges should not be neglected. First, there is no standard diagnosis criteria in China, so most patients in studies are diagnosed by clinical and image criteria. Second, all studies were retrospective. Therefore, some data were incomplete or inaccurate. Third, lacking a register system in China, some cases meet the included criteria may not be included. Forth, conclusion may not be generalizable to the other regions.

## Conclusion

Overall, this review summarizes studies and provides a up-to-date epidemiological features of SCI in China. Because the existence of limitations, it is challenging to obtain exact epidemiological data of SCI. Thus, more studies are needed to provide large amount of data and evidence. In addition, it is impeative to establish a national register system for SCI patients in the next few years and it will be helpful for throughtly understanding the epidemiological features of SCI in China.

## Author contributions

SY and ZS: study design, data analysis, original draft writing and editing. SY, ZS, FC, and JL: data collection, data analysis. SF: study design and supervision.

### Conflict of interest statement

The authors declare that the research was conducted in the absence of any commercial or financial relationships that could be construed as a potential conflict of interest.
